# Attitudes and expectations of primary care physicians regarding recreational cannabis legalization in Germany: a pre-implementation survey

**DOI:** 10.1186/s42238-025-00367-8

**Published:** 2025-12-02

**Authors:** Uta Hochheim, Frank Müller, Eva Maria Noack

**Affiliations:** 1 Department of General Practice, University Medical Center Göttingen, Humboldtallee 38, Göttingen, DE 37073 Germany; 2 Department of Family Medicine, College of Human Medicine, Michigan State University, Grand Rapids, MI USA

**Keywords:** Cannabis legalization, Physician attitudes, Primary care, Public health policy, Medical cannabis, Germany

## Abstract

**Background:**

On April 1st, 2024, Germany legalized recreational cannabis use under specific conditions. While policymakers extensively debated this change, the perspectives of healthcare providers who will address its consequences remain understudied. This study aims to characterize primary care physicians’ experiences with cannabis-consuming patients and their expectations regarding the effects of legalization.

**Methods:**

This is an exploratory cross-sectional survey among general practitioners (GPs) and practice-based anesthesiologists in three German federal states (Lower Saxony, Bavaria, and Saarland) from September 2023 to March 2024, preceding cannabis legalization. The 17-item questionnaire assessed physicians’ experiences with cannabis-consuming patients, medical cannabis prescribing practices, personal cannabis use experience, and expectations regarding legalization consequences.

**Results:**

Of 946 successfully delivered surveys, 239 physicians responded (25.3% response rate). Most physicians anticipated increased cannabis consumption and disorders post-legalization, with those providers with personal cannabis experience (37.9%) more optimistic about achieving policy goals such as cannabis quality control, compared to those without such experience. Despite 40.3% prescribing medical cannabis in their practice, respondents rarely screened specifically for cannabis use and only few showed interest in additional training.

**Conclusion:**

Our survey reveals that German primary care physicians anticipate increased cannabis consumption and patient inquiries following legalization, yet current screening practices remain limited with modest interest in additional training. Enhanced integration of healthcare providers into cannabis policy implementation could improve patient care and support public health objectives.

**Trial registration:**

NA

**Supplementary Information:**

The online version contains supplementary material available at 10.1186/s42238-025-00367-8.

## Background

On April 1 st 2024, Germany implemented a significant policy shift by legalizing the possession and consumption of cannabis for recreational use under specific conditions. This legislation allows adults to possess limited quantities for personal use while maintaining restrictions on public consumption and establishing regulatory frameworks for cultivation (Bundesministerium der Justiz 2024a). The change followed years of discussion and represents a major drug policy reform in Europe (Manthey et al. 2024).

Cannabis consumption is associated with variable health risks, that differ across consumption patterns and populations and include dependency disorders and risks for psychotic episodes (Hua 2021; Page et al. 2020). This applies particularly for adolescents and young adults. Cannabis consumption during adolescence negatively impacts academic performance, with associated increases in school dropout rates (Chan et al. 2024; Schaefer et al. 2021). Still, with approximately 10% of adult German residents reporting having used cannabis in the past 12 months, cannabis consumption has become more and more normalized over the last decades (Olderbak et al. 2024). Given this reality, a legalization of recreational use could reduce workload of the legal system, challenged with 3.1 cannabis-related criminal charges per 1,000 residents in 2021 (Manthey et al. 2025b). A legalization for recreational use could furthermore decrease racial enforcement disparities as indicated by US-based findings (Gunadi and Shi 2022).

In parallel with the increasing use of cannabis in Germany, restrictions on the prescription of medical cannabis were lifted in 2017. Since then, prescriptions have gained momentum. In the first half year of 2024, a total of 198,929 cannabis prescriptions covered by statutory health insurances were issued, accounting for a gross turnover of 105,210,979 EUR (GKV-Arzneimittel-Schnellinformation (GAmSi) 2024). This represents an increase compared to 2018 when there were 79,894 prescriptions generating a gross turnover of 30,823,067 EUR (GKV-Arzneimittel-Schnellinformation (GAmSi) 2018). A similar trend towards more medical cannabis prescriptions has been observed among privately insured Germans (approximately 11% of all residents (Jacke 2022). Notable prescribers included general practitioners (GPs) who accounted for 17% (Schmidt-Wolf and Cremer-Schaeffer 2021) to 25.8% (IQVIA Commercial GmbH & Co. OHG 2021) of all prescriptions, as well as outpatient anesthesiologists who accounted for 10.1% (IQVIA Commercial GmbH & Co. OHG 2021) to 49% (Schmidt-Wolf and Cremer-Schaeffer 2021) of all prescriptions, depending on the respective study. These specialists have developed clinical experience with cannabis as a therapeutic agent for conditions including chronic pain, spasticity, and tumor diseases (Gastmeier 2019).

Policymakers and public health officials have extensively debated the merits and risks of cannabis legalization. However, primary care physicians’ perspectives were notably absent from policy development discussions – despite their frontline role in managing medical cannabis patients and potentially being point of contact for questions on recreational use and addressing the consequences of problematic use–. Some opinions of physicians have been published as debate pieces (Fath 2022) prior to the legalization of recreational cannabis and the German Federal Medical Association (Bundesärztekammer) has opposed the new legislation (Deutscher Bundestag - Ausschuss für Gesundheit 2023; Deutsches Ärzteblatt 2024).

To address this knowledge gap and establish a baseline understanding of physician perspectives at this critical policy juncture, this study aims to: (1) characterize physicians’ experience with cannabis-consuming patients; (2) examine physicians’ experience and attitudes related to medical cannabis prescribing; (3) assess physician expectations regarding various consequences of cannabis legalization, including impacts on public health, patient behaviors, and clinical practice; (4) explore the relationship between physicians’ experience with cannabis, including personal cannabis use, and their professional perspectives/expectations regarding the consequences of legalization (5) analyze how sociodemographic factors and practice characteristics may influence these attitudes and expectations.

## Methods

This exploratory cross-sectional survey was conducted among primary care providers (GPs and office-based anesthesiologists) in three German federal states (Lower Saxony, Bavaria, and Saarland) from September 2023 to March 2024. The data collection period preceded Germany’s cannabis legalization on April 1, 2024. Results are reported following the Consensus-Based Checklist for Reporting of Survey Studies (CROSS reporting guidelines) on survey studies (Sharma et al. 2021).

### Survey development

A 17-item questionnaire was developed collaboratively among the author team, consisting of a GP, a sociologist, and an experienced health services researcher. We employed an inductive approach, beginning with a comprehensive review of relevant literature and official documents from the Federal Ministry of Health that outlined the intended health policy goals of cannabis legalization for recreational use. These policy goals included improving quality control of cannabis products, preventing distribution of contaminated substances, enhancing youth and consumer health protection, and reducing the black market for cannabis (Bundesministerium für Gesundheit (BMG) 2023, 2022; Sozialdemokratische Partei Deutschlands (SPD) et al. 2021).

Based on this review, we derived with a preliminary version of items that were grouped into five sections: (a) experiences with patients using cannabis, (b) experience and practice of prescribing medical cannabis in own practice, (c) personal cannabis use experience, (d) expectations regarding cannabis legalization consequences, and (e) sociodemographic and practice-related characteristics.

To allow standardized assessment, we developed statements that were to be rated on Likert scales. For items assessing personal opinions and attitudes where we anticipated a tendency to neutral responses, we used a 6-point Likert scale to urge respondents to indicate at least a slight tendency in either direction. For items regarding expected changes following legalization, we used 5-point scales to allow respondents to select “no change” as a midpoint option. Only the extreme values on these scales were labeled (e.g., “not at all” to “very frequently” or “strongly decrease” to “strongly increase”) to reduce potential anchoring effects.

The survey development process included multiple revision cycles within the research team to ensure content validity. Furthermore, we discussed preliminary versions with other health and social researchers at our department that were not part of the study team to receive feedback on clarity, used wording, and comprehensiveness. Before concluding the development process, we conducted pretesting with four GPs from Lower Saxony (Lenzner et al. 2016). During these pretests, participants completed the questionnaire while verbalizing their thought processes, followed by structured feedback sessions addressing question comprehension, response option appropriateness, and overall questionnaire flow. Participants were specifically asked to identify ambiguous wording, unclear instructions, or missing response options. With this feedback, we further refined and finalized our questionnaire. The questionnaire was developed in German to ensure cultural and contextual appropriateness for the target population. Both the original German version and an English forward-translation are included in the appendices A1 and A2 of this manuscript.

### Health care setting

In Germany, primary care is provided by a network of office-based physicians who operate as independent contractors within the statutory health insurance system. From the entire primary care physician workforce (*n* = 189,551, data from 2024), 29.3% and thus the largest group are GPs (Kassenärztliche Bundesvereinigung (KBV) 2025). These GPs provide general care, including management of urgent but not life-threatening conditions, continuous care also for people with chronic conditions, and preventive health services such as health check-ups and immunization. A key part of their work includes pain management for various acute and chronic conditions, such as the very common lower back pain, but also in more complicated care situation including cancer-related pain in end-of-life care. The majority of opioids and pain killers are prescribed through GPs (Glaeske 2022).

Unlike to many other countries where anesthesiologists primarily work in hospital settings, some anesthesiologists in Germany are working in practices (approximately 2.1% of the primary care physician workforce (Kassenärztliche Bundesvereinigung (KBV) 2025), where they mainly provide anesthesia as a service for day-clinics or outpatient practices, e.g. for teeth extraction and other ambulatory surgeries. Besides this, many provide specialized pain management for patients with chronic pain, which is one of the primary indications for medical cannabis. Therefore, office-based anesthesiologists represent an important group for medical cannabis prescribing in Germany. This reflects a structural feature of the German healthcare system where specialized ambulatory care, including complex pain management, occurs in office-based settings with direct patient access, rather than requiring hospital referrals.

Both providers, GPs and practice-based anesthesiologist are the main prescribers of cannabis in Germany and were thus addressed as target population in this study (Schmidt-Wolf and Cremer-Schaeffer 2021; IQVIA Commercial GmbH & Co. OHG 2021); Johnsen and Maag 2021).

### Sample and target population

The study population consisted of a purposively selected sample of GPs and office-based anesthesiologists. Potential participants were randomly identified from publicly accessible databases maintained by the Regional Associations of Statutory Health Insurance Physicians (Kassenärztliche Vereinigung) of the three federal states Lower Saxony, Bavaria, and Saarland. We employed block randomization to match federal state numbers of physicians and proportion of medical specialties in respective states (ratio of GPs/anesthesiologists on primary care workforce). Two cohorts of 1,000 physicians each were generated. Our methodological approach involved contacting the initial cohort and, contingent upon response rates, proceeding to the second cohort if necessary. The sample size determination was informed by previous physician surveys recently conducted in Germany, which reported response rates ranging from 2.9% (Beerheide 2022) or 6.4% (Radić et al. 2021) up to 14% (Zentralinstitut für die Kassenärztliche Versorgung in Deutschland (Zi) 2022) — notably lower than international averages. Based on these precedents, we anticipated approximately a 10% response rate, necessitating contact with potentially 2,000 physicians to achieve our target. Our objective was to include a minimum of 200 physicians, which would facilitate meaningful subgroup analyses of practitioners with and without cannabis-related experience (defined as treating cannabis-consuming patients, prescribing medical cannabis, or having personal cannabis use experience). As this threshold was reached in November, 2023, distribution of the questionnaire to the second cohort of 1,000 physicians was deemed unnecessary.

### Survey administration

The sample of 1,000 physicians were contacted by mail in September 2023. Participants received personalized invitation letters that explained the purpose of the survey and providing information on survey participation including information of data protection and data processing. By returning the completed survey, participants confirmed that they had understood the study purpose and consented to their responses being stored anonymously, evaluated, and used for the specified study purposes, including publications of results. Participants could respond by either returning the completed paper questionnaire using the provided prepaid envelope or by scanning a QR code to complete the survey online. In both cases, the survey was anonymous. The online survey was administered using LimeSurvey (LimeSurvey GmbH, Hamburg, Germany). Responses on paper-based questionnaires were manually entered into the LimeSurvey system upon receipt. To minimize human error in data entry, a second person checked the data. The survey was concluded March 31 st 2024 and no responses arrived later.

### Statistics

Descriptive statistics were used to characterize sample demographics and summarize the responses to all survey items. For categorical variables, we calculated frequencies and percentages; for continuous variables and Likert-scale items, we computed means and standard deviations. Response distributions for all Likert-scale items were visualized using histograms, showing the number of valid responses for each item. Missing values were excluded from analyses.

This descriptive part was followed by bivariate analyses to examine factors associated with physicians’ expectations regarding cannabis legalization. We investigated the influence of several key variables: personal cannabis experience (yes/no), medical specialty (GP vs. non-GP), medical cannabis prescribing status (prescriber vs. non-prescriber), gender, practice size (categorized by quarterly insurance claims), respondents’ age, and years in ambulatory practice. Non-parametric bivariate tests included Mann-Whitney *U* and Kruskal-Wallis tests. Associations between ordinal or continuous variables (age, years in practice) and physicians’ expectations were assessed using Spearman’s rho. For items that were relevant only to subgroups (such as expectations about changes in medical cannabis prescribing, which was only applicable to current prescribers), analyses were appropriately stratified and conducted only within the relevant subpopulation. Statistical significance was assumed at *p* <.05 for all analyses. All analyses were conducted using SPSS 30 (IBM Corp., Armonk, NY).

### Ethical considerations

The study protocol for this survey project was reviewed by the Ethics Research Board of University Medical Center Göttingen under application number 4/1/23. As this research included participants from further German Federal States, the study protocol and the initial vote was forwarded to responsible ethics committees of the Board of Physicians of Bavaria and Saarland and approved (Bavaria application number 2023 − 1094, Saarland application number 128/23).

## Results

### Sample characteristics

**Fig. 1:**
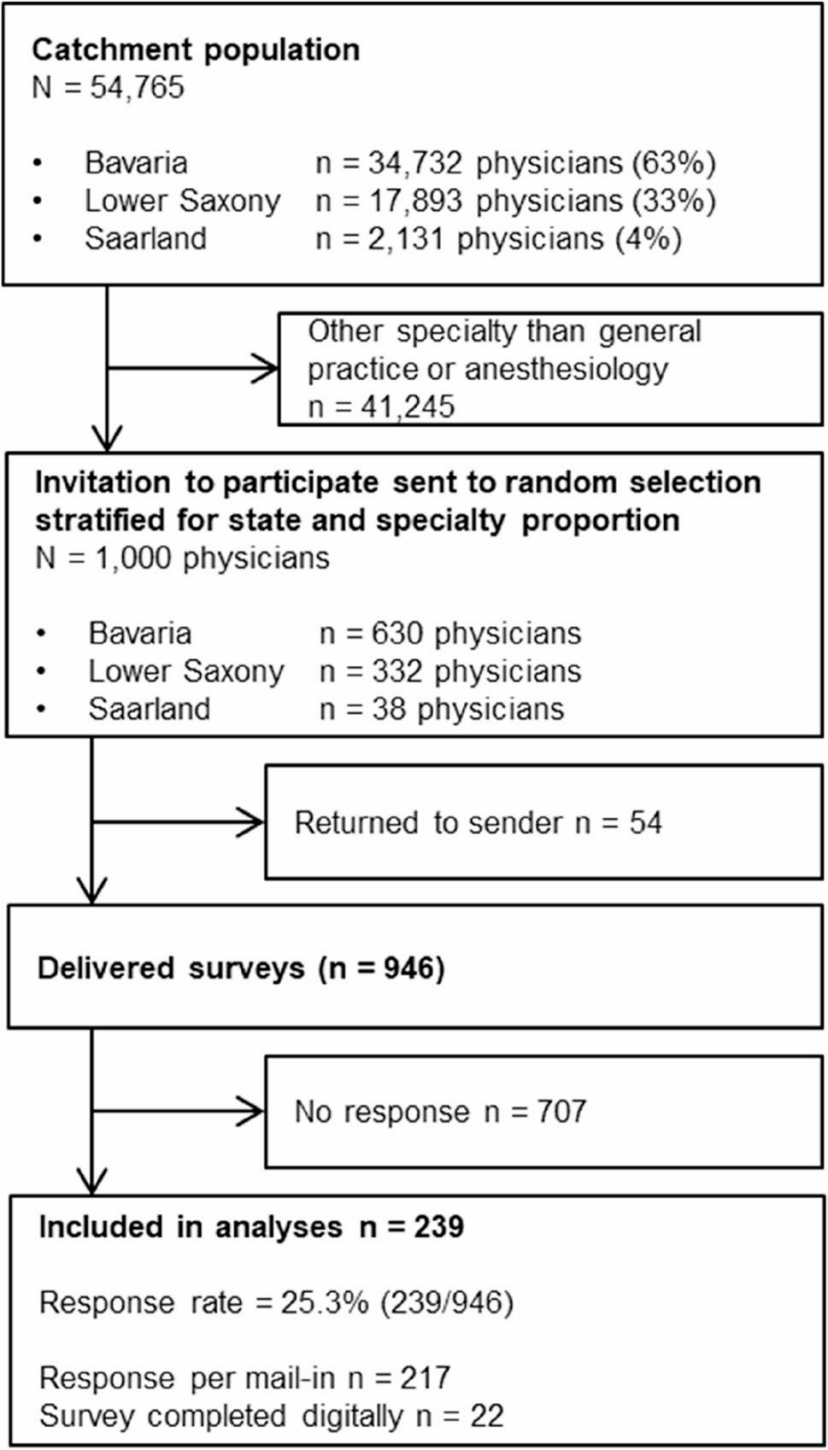
Flowchart of participant inclusion

Out of 13,520 general practitioners and anesthesiologists in the catchment area, we invited a random stratified sample of *n* = 1,000 that maintained the proportional distribution across the respective states. Of the 946 successfully delivered surveys, 239 were completed and included in the analyses, resulting in a response rate of 25.3%. The flowchart of participant inclusion is displayed in Fig. 1.

The sample primarily consisted of general practitioners (90.0%) with some anesthesiologists (11.7%) and physicians with additional other specialties (5.9%) (multiple answers were possible). These proportions correspond to the contacted physicians (GP 88.7%, anesthesiologists 11.3%). The majority of respondents were male (58.0%), with a mean age of 54.3 years (SD 10.2) and worked full-time (71.4%). Details of participant characteristics are presented in Table 1. Nearly all respondents (92.8%, *n* = 205) reported having patients who discuss cannabis with them.

**Table 1:** Characteristics of participants (*n* = 239)

Demographic Data		*n* (%)
Gender	Male	138 (58.0)
	Female	99 (41.6)
	Diverse	1 (0.4)
Age (years)	Mean (SD)	54.3 (10.2)
	30–44	43 (18.3)
	45–59	113 (48.1)
	60+	79 (33.6)
Professional Characteristics		
Medical Specialty (multiple responses permitted)	General Practice	215 (90.0)
	Anesthesiology	28 (11.7)
	Other specialty	14 (5.9)
Additional Board-certified Qualifications (multiple responses permitted)	Palliative Medicine	44 (18.4)
	Pain Medicine	17 (7.1)
	Emergency Medicine	39 (16.3)
	Acupuncture/Manual Medicine/Natural Medicine	38 (15.9)
	None	85 (35.6)
Years in Ambulatory Care	Mean (SD)	20.2 (11.0)
Working Hours per Week in Ambulatory Care	Full-time	170 (71.4)
	Part-time	68 (28.6)
Practice size (health insurance claims per quartal)	< 100 claims	7 (3.0)
	100–500 claims	29 (12.3)
	501-1,000 claims	77 (32.8)
	> 1,000 claims	122 (51.9)
Practice Type	Solo Practice	79 (33.2)
	Group Practice	123 (51.7)
	Practice Association	11 (4.6)
	Ambulatory Care Center	20 (8.4)
	Other	5 (2.1)

Missing data: Age (*n* = 4), Gender (*n* = 1), Years in Ambulatory Care (*n* = 1), Working Hours (*n* = 1), Patient Volume (*n* = 4), Practice Type (*n* = 1)

*SD* Standard deviation

### Physicians own experience with consumption of cannabis

Personal experience with cannabis use was reported by 37.9% (*n* = 89) of physicians. Of those with personal cannabis use history, the majority (57.1%) reported that they last used cannabis between ages 21 and 30, while 22.0% reported last using it between ages 11 and 20. A smaller proportion reported more recent use: 5.5% between ages 31–40, 6.6% between ages 41–50, 4.4% between ages 51–60, and 4.4% between ages 61–70.Physicians with personal cannabis experience were somewhat younger (mean age = 52.17 years) than those without such experience (mean age = 55.75 years; Mann-Whitney *U* = 5065.5, *p* =.015). There were no statistically significant differences in gender distribution, working hours, patient volume, practice type, or medical specialty between physicians with and without personal cannabis experience (all *p* >.05).

### Discussing cannabis consumption with patients

**Fig. 2:**
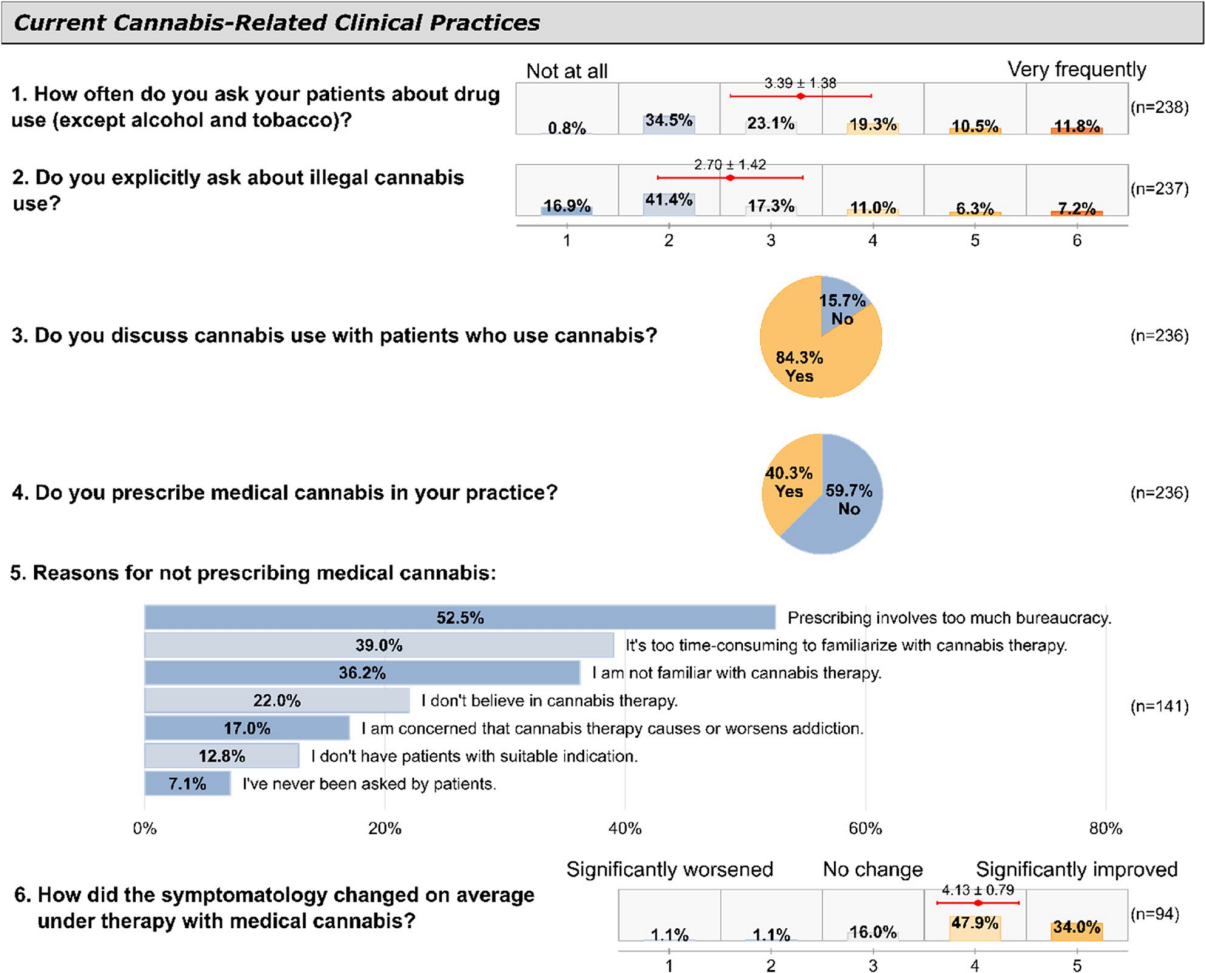
Responses on clinical practice and experience with medical cannabis

Physicians reported varying frequencies of inquiring about drug use (excluding alcohol and tobacco), as shown in Fig. 2. Non-GPs asked significantly more frequently (mean = 5.23 on a 6-point Likert scale from 1="not at all” to 6="very frequently”) than GPs (mean = 3.21, *p* <.001). This practice was not significantly associated with other physician characteristics including age, years in practice, personal cannabis experience, gender, or practice size (all *p* >.05).

Non-GPs also reported more specific inquiries about illegal cannabis use compared to GPs, though this difference was not statistically significant (means: 3.00 vs. 2.67 on the same 6-point scale, *p* =.310). Similarly, when physicians were asked specifically about inquiring about illegal cannabis use, the overall frequency was lower (mean = 2.70) than for general drug use inquiries, with 16.9% reporting they “never” asked specifically about cannabis. Neither gender, age, years in practice, nor personal experience with cannabis were associated with the frequency of specific cannabis use inquiries (all *p* >.05).

The most common occasions on which physicians ask their patients about drug use were if they suspected drug use (66%), had a gut feeling (41%), were taking an initial medical history (40%), or were conduction a health check-up (20%).

When asked to estimate how many patients they treat annually who use cannabis, 44.8% indicated they did not know. Among those providing estimates (*n* = 125), physicians reported a mean of 40.1 patients (SD = 53.2, median = 20, range = 1–300). Most physicians (84.3%) reported discussing cannabis use with patients who acknowledge consumption (Fig. 2).

### Medical cannabis prescribing practices

Of 236 respondents, 40.3% (*n* = 95) reported prescribing medical cannabis in their practice. The likelihood of prescribing medical cannabis was not associated with practice size, practice type, specialty (GP vs. non-GP), gender, or personal cannabis experience (all *p* >.05).

Among physicians who prescribed medical cannabis (*n* = 95), the most common indications were pain (69.5%), spasticity (15.8%), and other conditions (12.6%). Most prescribing physicians (81.9%) reported symptom improvement, while only 2.2% reported symptom worsening. When asked if patients receiving medical cannabis also consumed illegal cannabis, 44.2% reported none or few patients did so, while 7.4% reported many or very many did, though 38.9% were uncertain.

### Perspectives on legalization of recreational cannabis

**Fig. 3:**
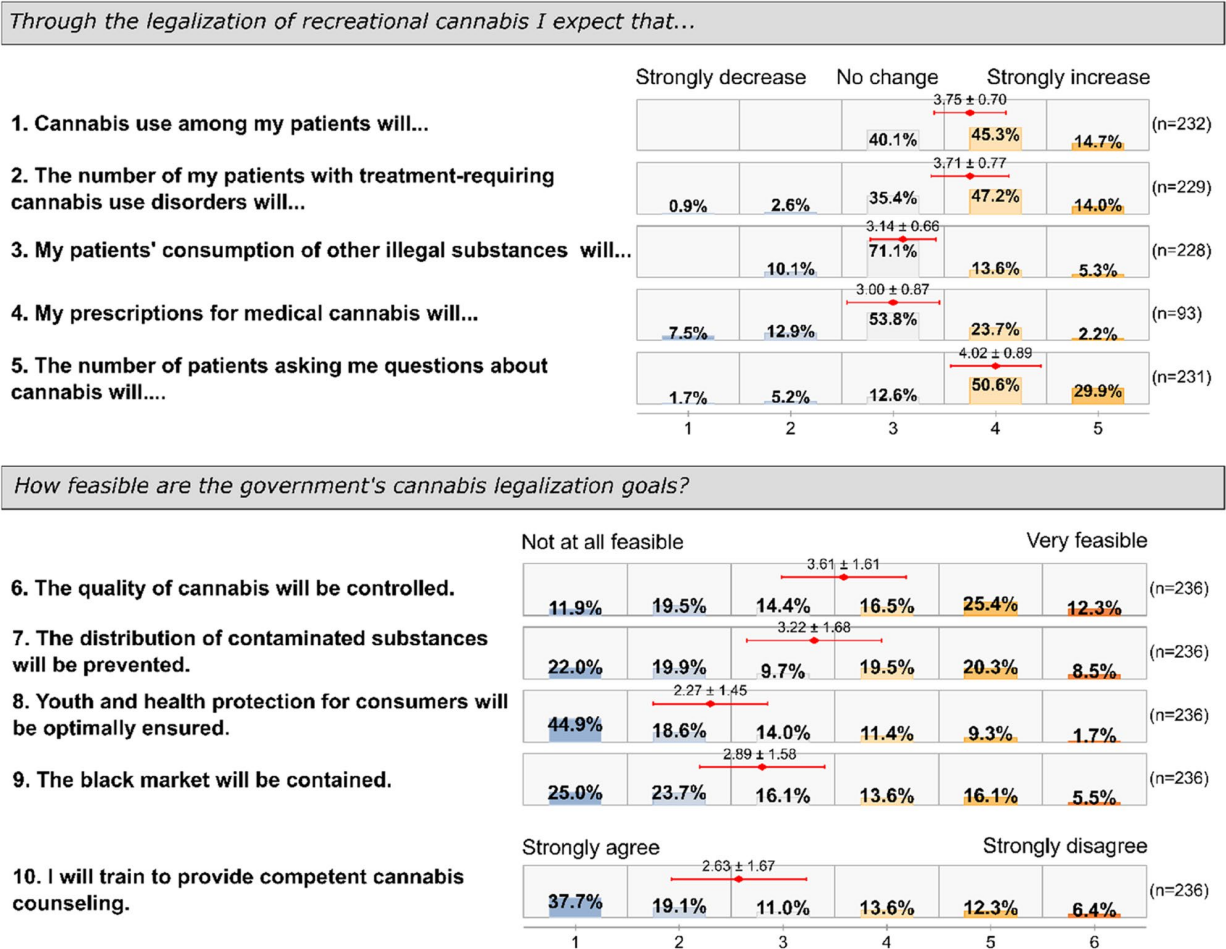
Responses on expectations towards legalization of recreational cannabis

Physicians were asked about their expectations regarding various consequences of cannabis legalization (rated on a 5-point Likert scale, 1="strongly decrease” to 5="strongly increase”), as well as their views on the feasibility of the governmental goals behind the new legislation (rated on a 6-point Likert scale, 1="not at all feasible” to 6="very feasible”). Fig. 3 presents an overview of their responses to these questions. Below, we examine each expectation area and the factors that influenced physicians’ perspectives.

Cannabis consumption among patients was generally expected to increase (mean = 3.75), with physicians who had no personal cannabis experience anticipating a greater increase (mean = 3.83) than those with personal cannabis experience (mean = 3.61, *p* =.024). No statistically significant differences were observed based on gender, practice size, specialty (GP vs. non-GP), or whether the physician prescribed medical cannabis.

Regarding patients with treatment-requiring cannabis use disorders (5-point scale), physicians anticipated an increase (mean = 3.71), with non-prescribers expecting a higher increase (mean = 3.81) than prescribers of medical cannabis (mean = 3.56, *p* =.040). No other statistically significant differences with other covariates were observed.

Most physicians expected minimal change in consumption of other illegal drugs (mean = 3.14), though non-prescribers anticipated more increase (mean = 3.21) than prescribers (mean = 3.03, *p* =.040). No other physician characteristics showed statistically significant associations with this expectation.

Among those who prescribe medical cannabis, expectations about changes in medical cannabis prescriptions were neutral (mean = 3.00). Male physicians expected more frequently a decline of own prescriptions than female physicians (mean = 3.30 vs. mean = 2.83, *p* =.038).

Physicians strongly expected increased patient inquiries about cannabis (mean = 4.02), with female physicians anticipating more questions (mean = 4.20) than male physicians (mean = 3.88, *p* =.027). Non-GPs expected fewer patient inquiries (mean = 3.68) compared to GPs (mean = 4.05, *p* =.031). No significant associations were found with other physician characteristics.

When assessing the feasibility of policy goals on a 6-point Likert scale, physicians with personal cannabis experience showed greater optimism about quality control (mean = 3.84) than those without such experience (mean = 3.46, *p* =.066). Male physicians also showed greater optimism about quality control (mean = 3.83) than female physicians (mean = 3.30, *p* =.012).

Physicians with personal cannabis experience expressed greater confidence in preventing contaminated substance distribution (mean = 3.55) compared to those without such experience (mean = 3.00, *p* =.017).

Physicians were generally skeptical about youth protection measures (mean = 2.27), though those with personal cannabis experience (mean = 2.54) were slightly less skeptical than those without (mean = 2.10, *p* =.030).

Cannabis-experienced physicians were more optimistic about black market reduction (mean = 3.17) compared to those without such experience (mean = 2.71, *p* =.037). Physicians who prescribed medical cannabis (mean = 2.96) expressed greater likelihood of pursuing cannabis counseling education following legalization compared to non-prescribers (mean = 2.40, *p* =.003). No other significant associations were observed.

## Discussion

Our survey reveals that GPs and office-based anesthesiologists expect recreational cannabis legalization in Germany to increase both consumption and treatment-requiring cannabis use disorders among their patients. While most of physicians anticipate more patient inquiries about cannabis use, only a minority expressed willingness to pursue training and education on this topic. Personal cannabis experience, reported by 37.9% of respondents, was associated with more optimistic views regarding achievement of proposed policy goals such as quality control and black market reduction.

Responding physicians rarely screen specifically for cannabis use, with 16.9% indicating “never asking” and a mean frequency of only 2.7 on a 6-point scale. This is particularly concerning given that approximately 4.5 million Germans (8.8% prevalence) use cannabis, with 1.3 million experiencing problematic use (Rauschert et al. 2022), and inpatient cases for cannabis-related disorders have increased 4.8-fold between 2000 and 2018 (Gahr et al. 2022) and even accelerated more during early COVID-19 pandemic (Olderbak et al. 2024). Our data suggest that cannabis use remains largely “off the radar” for many primary care physicians, who are likely not systematically screening for a potentially hazardous habit that is pursued by nearly one in eleven Germans. In Canada, where cannabis was legalized in 2018 on national level, a study analyzing electronic medical record in primary care in 2019 showed that very few patients had cannabis use recorded suggesting that health care providers did not routinely or systematically screen for cannabis use (Soos et al. 2023). This screening gap contrasts with guidelines, such as the Preventive Services Task Force (USPSTF) that recommends universal screening for unhealthy drug use, including cannabis, in all adults 18 years and older (Sazegar 2021). The guidelines of the German Joint Federal Committee on preventive screening examinations which are available to any adult with statutory health insurance (so-called “health check-ups”) reveal an inconsistency: While the medical history section mentions only nicotine and alcohol abuse as personal risk factors to be assessed, the subsequent risk-adapted medical advice explicitly includes education on nicotine, alcohol and drug consumption (Gemeinsamer Bundesausschuss (G-BA) 2019). This suggests that drug use screening was implicitly intended to be part of the medical history assessment but was inadvertently omitted from the medical history section of the guidelines. The higher screening frequency among anesthesiologists in this survey may reflect preoperative risk evaluation (Zöllner et al. 2024).

A considerable proportion of responding physicians had personal cannabis experience (37.9%) mostly during young adulthood. It is interesting that they disclosed the activity, which was illegal at the time of the survey. The anonymity of the survey may have contributed to respondents disclosing their own cannabis use quite openly. Given the persistent stigma surrounding cannabis use, the actual prevalence may still be underestimated. However, our data on self-use is consistent with the 2021 Epidemiological Survey on Addiction (Epidemiologischer Suchtsurvey) which reported a lifetime prevalence of 34.7% among 18–64 year old Germans. Percentage was higher among males (38.9%) than among females (30.2%) (Deutsche Hauptstelle für Suchtfragen e.V. 2025). As our sample includes 58.0% male respondents, this might explain the slight above-national average finding.

Literature has indicated that physicians with cannabis experience have more positive attitudes toward medical cannabis (Syed et al. 2025; HaGani et al. 2022; Zammit Dimech et al. 2025). While own cannabis use experience could potentially facilitate more empathetic and informed patient counseling, it raises questions about potential bias in clinical decision-making, e.g. in prescribing medical cannabis or not.

While international guidelines emphasize the crucial role of family physicians in cannabis screening and prevention (Page et al. 2020; Sazegar 2021), the German combined legalization framework is barely integrating primary care providers into prevention and counseling strategies. The German approach mainly focuses on structural measures such as neutral packaging, child-proof containers, consumption bans near schools and playgrounds, and expands existing prevention programs in various community settings including schools, vocational schools, social media, youth welfare facilities, institutions for cognitively impaired individuals, sports clubs, and workplaces (Bundesministerium für Gesundheit (BMG) 2022). This is particularly relevant given that physicians in our cohort expected increased patient inquiries about cannabis but a substantial proportion of respondents are unwilling to educate themselves on cannabis use. Assessing physicians’ competencies in counseling, and management of cannabis-related health problems was not scope of this study. However, a potential disconnect between the lack of preparedness and the anticipated clinical demands raises concerns about potential gaps in the public health response as it is assumed that effective cannabis prevention requires active involvement of healthcare providers (Kourgiantakis et al. 2023).

Our findings reveal that only 40.3% of surveyed physicians had prescribed medical cannabis, which may reflect the complex bureaucratic requirements associated with controlled substance prescriptions prior to the recent legislative changes. Before 2017, medical cannabis in Germany required exceptional authorization from federal authorities. Since 2017, a new law allowed physicians to prescribe medical cannabis products via narcotic prescriptions (Cremer-Schaeffer and Knöss 2019; Bundesministerium der Justiz und für Verbraucherschutz 2017). The Medical Cannabis Act (MedCanG) implemented on April 1, 2024, removed most cannabis medications from narcotic drug classification, enabling prescription via standard or electronic prescriptions (Bundesministerium der Justiz 2024b). Indeed, excessive administrative burden was frequently cited in this survey as a deterrent to cannabis prescribing. Concurrently, we identified a substantial proportion of physicians who are unfamiliar with cannabis therapeutics. Physicians’ experience of a lack of knowledge is in line with systematic literature reviews(Rønne et al. 2021; Gardiner et al. 2019) but it contrasts with a recently published market research survey in which physicians, including GPs, ranged themselves quite knowledgeable about cannabis based products in healthcare (Russo et al. 2024). A qualitative interview study from Germany among patients receiving medical cannabis showed that also patients perceived knowledge gaps among healthcare providers (Borojevic and Söhner 2025). Health care trainees lack knowledge about medical cannabis (Zolotov et al. 2021) and the necessity for respective adequate training has been highlighted (Syed et al. 2025). A recently published consensus statement lists six core competencies on medical cannabis for medical education curricula (Zolotov et al. 2025). These competencies would qualify healthcare providers not only to safely integrate therapeutic cannabis applications into health management but also to pursue approaches to reduce (problematic) use.

The future will show whether patients previously receiving medical cannabis turn to recreational use (e.g., home cultivation), a phenomenon documented in North American literature (Armstrong 2024; Boehnke et al. 2024). Interestingly, most physicians in our study do not anticipate such shifts, indicating that their prescriptions for medical cannabis will not change. It is noteworthy that physicians who prescribe medical cannabis generally report positive patient outcomes, which aligns with patient-reported experience studies (Tait et al. 2023; Dickinson et al. 2025) and studies on physicians experiences with patients receiving medical cannabis (Rønne et al. 2021; Zolotov et al. 2018) but contrasts with numerous interventional trials that demonstrate minimal or no benefit across various indications (Petzke et al. 2016; Wang et al. 2021; Inglet et al. 2020). This divergence between positive real-world reports and minimal benefits in interventional trials is likely due to a combination of placebo effects, patient self-selection bias in observational reports, and the standardizations of products in randomized controlled trials compared to real-world personalized dosing.

Regarding the impact of legalization on their patients, respondents predominantly expressed neutral to negative expectations: most anticipate increased cannabis consumption and cannabis use disorders. Other countries that have legalized cannabis for non-medical purposes, have seen an increase of cannabis use that follows a pre-existing trend (Goodman et al. 2024). However, respondents project minimal influence on the use of other substances. This suggests that the traditional conceptualization of cannabis as a “gateway drug” to harder substances is not common among the surveyed physicians.

Concerning the objectives of cannabis legalization of the German Federal Government (Bundesministerium für Gesundheit (BMG) 2023, 2022; Sozialdemokratische Partei Deutschlands (SPD) et al. 2021), physicians expressed predominantly pessimistic views, albeit with considerable heterogeneity in responses. Youth protection and health promotion goals were most clearly perceived as unlikely to be achieved. The effect of legalization on young people’s cannabis use are unclear as the differences between pre- and post-legalization consumption may also be due to pre-existing trends rather than to policy change(Ladegard et al. 2020) or because of increased self-report after legalization (Cerdá et al. 2020). First evaluations of the partial legalization of cannabis in Germany have shown so far no impact on cannabis consumption among young people (Manthey et al. 2025a). In Canada, cannabis consumption has increased in people older than 25 years following three years after legalization. Results for younger people are mixed, probably showing that the pre-legalization trend of a decrease has been reversed (Rubin-Kahana et al. 2022; Hall et al. 2023). Similarly critical, though somewhat more varied, were physicians’ attitudes regarding the elimination of black market activity. Somewhat contradictorily, respondents expressed cautious optimism that legalization might improve quality control of cannabis products and reduce harmful adulterants. These objectives were probably deemed the most potentially achievable among the legalization goals.

### Limitations and strengths

This study comes with limitations. First, due to the cross-sectional design it provides only a snapshot of physician attitudes immediately prior to cannabis legalization. Second, our study relies on self-reported data that may be subject to a social desirability and/or recall bias, particularly regarding questions about personal cannabis use or professional practices in substance use screening. Third, there might be a selection bias as physicians with stronger opinions may have been more prone to participate, which might have skewed results toward more polarized perspectives. Fourth, our sample from three federal states may not fully represent the diversity of physician attitudes across Germany, particularly given the potential regional variations in cannabis use patterns (Been et al. 2016). The three federal states included in this survey represent distinct sociocultural contexts (i.e. patient demographics, and historical enforcement approaches to cannabis) that may have influenced physician responses. Bavaria is known for strict drug enforcing policies (Bayerisches Staatsministerium für Gesundheit, Pflege und Prävention (StMGP) 2025), while Lower Saxony represents a mix of urban and rural areas with more moderate attitudes and a border to the Netherlands, where recreational cannabis has been legal for decades. Saarland is as a smaller border state with proximity to France and Luxembourg with stricter regulations. To keep anonymity, respondents’ federal state was not inquired in the survey. Therefore, we cannot analyze the data at the federal state level. Future multi-state studies should include an eastern federal state and a city state such as Berlin, the federal state with the highest cannabis use rates (Olderbak et al. 2023). Future studies, including qualitative approaches, could assess how regional policy traditions, urbanization levels, and local cannabis prevalence rates influence physician attitudes. Fifth, our focus on GPs and office-based anesthesiologists anesthesiologists as the specialties that most frequently prescribe medical cannabis, excludes specialties such as neurologists and oncologists who prescribe for specific indications and may hold different perspectives shaped by their patient populations and therapeutic contexts. Thus, the results cannot be inferred across all medical cannabis contexts. Lastly, the questionnaire, though pretested, has not undergone comprehensive validation, which may affect the reliability and validity of the measures. In absence of existing validated instruments, we developed a brief, pragmatic questionnaire focused on content validity. While pretest feedback confirmed item clarity and relevance, the lack of formal validation testing means we cannot verify the reliability or factorial structure of our measures. Despite these limitations, our study offers several strengths: Data collection coincided with the legislative procedure, so physicians were “exposed” to the ongoing discourse and have likely developed an opinion towards legalization of recreational cannabis. This also might have contributed to the high response rate. Our study established a pre-implementation baseline from three rather diverse German Federal States which can be used for further research on acceptance and cannabis-related prescribing and screening practices in the future.

## Conclusions

Our survey reveals that German primary care physicians anticipate increased cannabis consumption and more patient inquiries following legalization, yet current cannabis screening practices remain limited with only modest interest in pursuing additional training. The current public health approach has not systematically engaged primary care providers in cannabis-related prevention and counseling strategies. These findings suggest that enhanced integration of healthcare providers into cannabis policy implementation could improve patient care and support achievement of public health objectives. These baseline findings will be valuable for pre-post comparisons. Follow-up studies can assess whether physician expectations align with actual post-legalization outcomes, e.g. whether physicians’ expectations regarding patient inquiries, cannabis consumption patterns, and use disorders have occurred. Research investigating whether knowledge and training gaps affect the quality of patient care can help improve the training of physicians and healthcare delivery.

## Supplementary Information

Supplementary Material 1.

Supplementary Material 2.

## Data Availability

The datasets used and analyzed during the study are not publicly available but can be obtained from the authors upon reasonable request within a data sharing agreement.

## Abbreviations

BMG: Bundesministerium für Gesundheit (Federal Ministry of Health)
CanG: Cannabisgesetz (Cannabis Law)
CROSS: Consensus-Based Checklist for Reporting of Survey Studies
GAmSi: GKV-Arzneimittel-Schnellinformation (Statutory Health Insurance Medication Information)
GKV: Gesetzliche Krankenversicherung (Statutory Health Insurance)
GPs: General Practitioners
KBV: Kassenärztliche Bundesvereinigung (National Association of Statutory Health Insurance Physicians)
KCanG: Konsumcannabisgesetz (Cannabis Consumption Act)
MedCanG: Medical Cannabis Act
SD: Standard Deviation
SPD: Sozialdemokratische Partei Deutschlands (Social Democratic Party of Germany)
USPSTF: US Preventive Services Task Force

## References

[CR1] Armstrong MJ. Canada’s recreational cannabis legalization and medical cannabis patient activity, 2017–2022. Am J Public Health. 2024;114(8):S673-80. 10.2105/AJPH.2024.307721. 39361903 10.2105/AJPH.2024.307721 PMC11499694

[CR2] Bayerisches Staatsministerium für Gesundheit, Pflege und Prävention (StMGP). Grundsätze der Bayerischen Staatsregierung zu Sucht und Drogen. Beschluss der Bayerischen Staatsregierung vom 13. Mai 2025. Hg. v. Bayerische Staatsregierung. München; 2025. (Available online, last accessed: 05.11.2025). https://www.stmgp.bayern.de/wp-content/uploads/2025/05/2025_05_13_preprint_grundsaetze_der_bayerischen_staatsregierung_zu_sucht_und_drogen.pdf.

[CR3] Been F, Bijlsma L, Benaglia L, Berset J-D, Botero-Coy AM, Castiglioni S et al. Assessing geographical differences in illicit drug consumption–A comparison of results from epidemiological and wastewater data in Germany and Switzerland. In: Drug and alcohol dependence 161, S. 2016:189–199. 10.1016/j.drugalcdep.2016.02.002. 10.1016/j.drugalcdep.2016.02.002 26896168

[CR4] Beerheide R. Klimawandel: Die it Abstand größte Krise. Deutsches Ärzteblatt. 2022;119(45):–8054.

[CR5] Boehnke KF, Sinclair R, Gordon F, Hosanagar A, Roehler DR, Smith T, et al. Trends in U.S. medical cannabis registrations, authorizing clinicians, and reasons for use from 2020 to 2022. Ann Intern Med. 2024;177(4):458–66. 10.7326/M23-2811. 38588545 10.7326/M23-2811 PMC11614148

[CR6] Borojevic V, Söhner F. Detecting and understanding potential stigma among medical cannabis users in Germany. BMC Public Health. 2025;25(1):874. 10.1186/s12889-025-22084-w. 40045288 10.1186/s12889-025-22084-w PMC11884029

[CR7] Bundesministerium der Justiz. Gesetz zum Umgang Mit Konsumcannabis (Konsumcannabisgesetz - KCanG). 2024a. Last accessed: 02.04.2025. https://www.gesetze-im-internet.de/kcang/BJNR06D0B0024.html.

[CR8] Bundesministerium der Justiz. Gesetz zur Versorgung mit Cannabis zu medizinischen und medizinisch-wissenschaftlichen Zwecken (Medizinal-Cannabisgesetz - MedCanG). 2024b. (Available online, last accessed: 19.05.2025.) https://www.gesetze-im-internet.de/medcang/BJNR06D0C0024.html.

[CR9] Bundesministerium der Justiz und für Verbraucherschutz. Gesetz zur Änderung betäubungsmittelrechtlicher und anderer Vorschriften vom 06. März 2017, BGBl I, S 403#. In: *Bundesgesetzblatt* (Teil I Nr. 11). Online verfügbar unter. 2017. https://www.bgbl.de/xaver/bgbl/start.xav?startbk=Bundesanzeiger_BGBl&start=//*%5b@attr_id=%27bgbl117s0403.pdf%27%5d#/switch/tocPane?_ts=1747647175738, zuletzt geprüft am 19.05.2025.

[CR10] Bundesministerium für Gesundheit (BMG). Eckpunktepapier der Bundesregierung zur Einführung einer kontrollierten Abgabe von Cannabis an Erwachsene zu Genusszwecken. 2022. (Available online, last accessed: 24.04.2025.) https://www.bundesgesundheitsministerium.de/fileadmin/Dateien/3_Downloads/Gesetze_und_Verordnungen/GuV/C/Kabinettvorlage_Eckpunktepapier_Abgabe_Cannabis.pdf.

[CR11] Bundesministerium für Gesundheit (BMG). Entwurf eines Gesetzes zum kontrollierten Umgang mit Cannabis und zur Änderung weiterer Vorschriften (Cannabisgesetz – CanG). Bundesregierung; 2023. Available online, last accessed: 24.04.2025. https://www.bundesgesundheitsministerium.de/fileadmin/Dateien/3_Downloads/C/Cannabis/Gesetzentwurf_Cannabis_Kabinett.pdf.

[CR12] Cerdá M, Mauro C, Hamilton A, Levy NS, Santaella-Tenorio Julián, Hasin D, et al. Association between recreational marijuana legalization in the united States and changes in marijuana use and cannabis use disorder from 2008 to 2016. JAMA Psychiatry. 2020;77(2):165–71. 10.1001/jamapsychiatry.2019.3254. 31722000 10.1001/jamapsychiatry.2019.3254 PMC6865220

[CR13] Chan O, Daudi A, Ji D, Wang M, Steen JP, Parnian P, et al. Cannabis use during adolescence and young adulthood and academic achievement: a systematic review and meta-analysis. JAMA Pediatr. 2024;178(12):1280–9. 10.1001/jamapediatrics.2024.3674. 39374005 10.1001/jamapediatrics.2024.3674 PMC11459363

[CR14] Cremer-Schaeffer P, Knöss W. Cannabis zu medizinischen Zwecken – Das Gesetz vom März 2017 und seine Vorgeschichte. Bundesgesundheitsbl. 2019;62(7):801–5. 10.1007/s00103-019-02962-6. 10.1007/s00103-019-02962-6 31139838

[CR15] Deutsche Hauptstelle für Suchtfragen e.V, Herausgeber. DHS jahrbuch sucht 2025. Cannabis- Zahlen und Fakten. Unter mitarbeit von Eva Hoch, Heiko Bergmann, Elena gommes de Mato, Justin Möckl, Monika Murawski, Franziska Schneider et al. Deutsche Hauptstelle für Suchtfragen e.V. Hamm: Pabst Science; 2025.

[CR16] Deutscher Bundestag - Ausschuss für Gesundheit. Stellungnahme der Bundesärztekammer zum Entwurf eines Gesetzes der Bundesregierung zum kontrollierten Umgang mit Cannabis und zur Änderung weiterer Vorschriften (Cannabisgesetz – CanG) BT-Drs. 20/8704) und zum Antrag der Fraktion der CDU/CSU „Cannabislegalisierung stoppen, Gesundheitsschutz verbessern – Aufklärung, Prävention und Forschung stärken (BT-Drs. 20/8735). Ausschussdrucksache. 2023;20(14)154(11). Available online, last accessed: 13.05.2025. https://www.bundestag.de/resource/blob/974438/821ee52941b81ab9ee546a780efbb224/20_14_0154-11-_Bundesaerztekammer_Cannabis.pdf.

[CR17] Deutsches, Ärzteblatt, editors. Mediziner enttäuscht über Entscheidung zu Cannabis. 2024. Available online, last accessed: 23.02.2024, zuletzt geprüft am 13.05.2025. https://www.aerzteblatt.de/news/mediziner-enttaeuscht-ueber-entscheidung-zu-cannabis-0825fb26-dfcb-462f-abce-7d796e61dfa9.

[CR18] Dickinson Mary, Erridge Simon, Warner-Levy John, Clarke Evonne, McLachlan Katy, Coomber Ross, et al. UK medical cannabis registry: an analysis of outcomes of medical cannabis therapy for hypermobility-associated chronic pain. ACR Open Rheumatol. 2025;7(3):e70024. 10.1002/acr2.70024. 40079426 10.1002/acr2.70024 PMC11905011

[CR19] Fath R. Führt die Legalisierung von Cannabis ur analisierung des medizinischen Einsatzes? MMW Fortschr Med. 2022;164(4):65. 10.1007/s15006-022-0866-3. 35211917 10.1007/s15006-022-0866-3

[CR20] Gahr M, Ziller J, Keller F, Muche R, Preuss UW, Schönfeldt-Lecuona C. Incidence of inpatient cases with mental disorders due to use of cannabinoids in germany: a nationwide evaluation. Eur J Public Health. 2022;32(2):239–45. 10.1093/eurpub/ckab207. 35043164 10.1093/eurpub/ckab207 PMC8975525

[CR21] Gardiner KM, Singleton JA, Sheridan J, Kyle GJ, Nissen LM. Health professional beliefs, knowledge, and concerns surrounding medicinal cannabis - a systematic review. PLoS One. 2019;14(5):e0216556. 10.1371/journal.pone.0216556. 31059531 10.1371/journal.pone.0216556 PMC6502454

[CR22] Gastmeier Knud. Einsatz von Cannabisarzneimitteln in der Schmerz- und Palliativmedizin. Schmerz. 2019;33(5):408–14. 10.1007/s00482-019-00406-3. 31444575 10.1007/s00482-019-00406-3

[CR23] Gemeinsamer Bundesausschuss (G-BA). Richtlinie des Gemeinsamen Bundesausschusses über die Gesundheitsuntersuchungen zur Früherkennung von Krankheiten (Bundesanzeiger BAnz AT, 06.03.2020 B2). 2019. Available online https://www.g-ba.de/downloads/62-492-2383/GU-RL_2020-11-20_iK-2021-02-12.pdf.

[CR24] GKV-Arzneimittel-Schnellinformation (GAmSi). Sonderbeilage zur GKV-Arzneimittel-Schnellinformation für Deutschland nach § 84 Abs. 5 SGB V. Bruttoumsätze und Verordnungen von Cannabinoidhaltigen Fertigarzneimitteln und Zubereitungen von Januar bis Juni 2018. Hg. v. Spitzenverband Bund der Krankenkassen (GKV-Spitzenverband). Berlin; 2018. last accessed: 03.11.2025. https://www.gkv-gamsi.de/media/dokumente/quartalsberichte/2018/q2_18/Bundesbericht_GAmSi_201806_konsolidiert_Sonderbeilage_Cannabis.pdf.

[CR25] GKV-Arzneimittel-Schnellinformation (GAmSi). Sonderbeilage zur GKV-Arzneimittel-Schnellinformation für Deutschland nach § 84 Abs. 5 SGB V. Bruttoumsätze und Verordnungen von Cannabinoidhaltigen Fertigarzneimitteln und Zubereitungen von Januar bis Juni 2024. Hg. v. Spitzenverband Bund der Krankenkassen (GKV-Spitzenverband). Berlin; 2024. Available online, last accessed: 13.05.2025. https://www.gkv-gamsi.de/media/dokumente/quartalsberichte/2024/q2_29/Bundesbericht_GAmSi_202406_konsolidiert_Sonderbeilage_Cannabis.pdf.

[CR26] Glaeske G. Opioidreport 2022. Unter Mitarbeit von Andrea Ernst, Friederike Höfel, Elisabeth Horn, Mona Lorenz, Julia Misonow, Lutz Muth et al. Universität Bremen, SOCIUM. Bremen; 2022. Available online (last accessed: 13.05.2025) https://www.hkk.de/fileadmin/dateien/allgemeines_uebergeordnet/reports/gesundheitsreports/2022_hkk_gesundheitsreport_opioide_web.pdf.

[CR27] Goodman Samantha, Dann Matthew J., Fataar Fathima, Abramovici Hanan. How have cannabis use and related indicators changed since legalization of cannabis for non-medical purposes? Results of the Canadian Cannabis Survey 2018–2022. Int J Drug Policy. 2024;127:104385. 10.1016/j.drugpo.2024.104385. 38520961 10.1016/j.drugpo.2024.104385

[CR28] Gunadi Christian, Shi Yuyan. Cannabis decriminalization and racial disparity in arrests for cannabis possession. Soc Sci Med. 2022;293:114672. 10.1016/j.socscimed.2021.114672. 34954673 10.1016/j.socscimed.2021.114672 PMC9170008

[CR29] HaGani Neta, Sznitman Sharon, Dor Michael, Bar-Sela Gil, Oren Dana, Margolis-Dorfman Lilia, et al. Attitudes toward the use of medical cannabis and the perceived efficacy, side-effects and risks: a survey of patients, nurses and physicians. J Psychoactive Drugs. 2022;54(5):393–402. 10.1080/02791072.2021.2009598. 34893011 10.1080/02791072.2021.2009598

[CR30] Hall Wayne, Stjepanović Daniel, Dawson Danielle, Leung Janni. The implementation and public health impacts of cannabis legalization in Canada: a systematic review. Addiction (Abingdon, England). 2023;118(11):2062–72. 10.1111/add.16274. 37380613 10.1111/add.16274 PMC10953418

[CR31] Hua Liwei L. Collaborative care in the identification and management of psychosis in adolescents and young adults. Pediatrics. 2021. 10.1542/peds.2021-051486. 34031232 10.1542/peds.2021-051486

[CR32] Inglet S, Winter B, Yost SE, Entringer S, Lian A, Biksacky M, et al. Clinical data for the use of cannabis-based treatments: a comprehensive review of the literature. Ann Pharmacother. 2020;54(11):1109–43. 10.1177/1060028020930189. 32483988 10.1177/1060028020930189

[CR33] IQVIA Commercial GmbH & Co. OHG. IQVIA Flashlight. Unter Mitarbeit von Gisela Maag. Frankfurt am Main (88); 2021. Available online https://www.iqvia.com/-/media/iqvia/pdfs/germany/flashlight/flashlight-88-iqvia-122021.pdf.

[CR34] Jacke CO. Verordnung cannabinoidhaltiger Arzneimittel in Deutschland unter besonderer Berücksichtigung der Privatversicherten (2017–2020). WIP-Kurzanalyse Juni 2022. Hg. v. Wissenschaftliches Institut der PKV. 2022. Available online, last accessed: 13.05.2025. https://www.wip-pkv.de/fileadmin/DATEN/Dokumente/WIP-Kurzanalysen/WIP-Kurzanalyse-Verordnung_cannabinoidhaltiger_Arzneimittel_in_Deutschland.pdf.

[CR35] Johnsen EØ, Maag G. Medizinal-Cannabis. Markt und ersorgung im Jahr 2020. Pharm Ind. 2021;83(6):752–62. Last accessed: 13.05.2025. https://www.iqvia.com/-/media/iqvia/pdfs/germany/publications/artikel-in-der-fachpresse/iqvia-artikel-medizinal-cannabis-pharmind-0621.pdf.

[CR36] Kassenärztliche Bundesvereinigung (KBV). Gesundheitsdaten. Mehr Ärztinnen und Ärzte, aber kürzere Arbeitszeiten. 2025. Available online, last accessed: 24.04.2025. https://gesundheitsdaten.kbv.de/cms/html/16393.php.

[CR37] Kourgiantakis T, Lee E, Kosar A, Kumsal Tekirdag; Tait C, Lau CKY, McNeil S, et al. Youth cannabis use in Canada post-legalization: service providers’ perceptions, practices, and recommendations. Subst Abuse Treat Prev Policy. 2023;18(1):36. 10.1186/s13011-023-00550-1. 37349741 10.1186/s13011-023-00550-1 PMC10288694

[CR38] Ladegard K, Thurstone C, Rylander M. Marijuana legalization and youth. Pediatrics. 2020;145(Suppl 2):S165–74. 10.1542/peds.2019-2056D. 32358207 10.1542/peds.2019-2056D

[CR39] Lenzner T, Neuert C, Otto W. Kognitives pretesting. GESIS Survey Guidelines; 2016.

[CR40] Manthey J, Jacobsen B, Kalke J, Kraus L, Radas S, Schranz A et al. Evaluation des Konsumcannabisgesetzes (EKOCAN): 1. Zwischenbericht. Hamburg. 2025a.

[CR41] Manthey J, Klinger S, Rosenkranz M, Schwarzkopf L. Cannabis use, health problems, and criminal offences in germany: National and state-level trends between 2009 and 2021. Eur Arch Psychiatry Clin NeuroSci. 2025b;275(2):555–64. 10.1007/s00406-024-01778-z. 38502205 10.1007/s00406-024-01778-z PMC11910392

[CR42] Manthey Jakob, Rehm Jürgen, Verthein Uwe. Germany’s cannabis act: a catalyst for European drug policy reform? The Lancet Regional Health – Europe. 2024. 10.1016/j.lanepe.2024.100929. 38779298 10.1016/j.lanepe.2024.100929 PMC11109464

[CR43] Olderbak S, Möckl J, Manthey J, Lee S, Rehm Jürgen, Hoch E, Kraus L. Trends and projection in the proportion of (heavy) cannabis use in Germany from 1995 to 2021. Addiction (Abingdon England). 2024;119(2):311–21. 10.1111/add.16356. 37816631 10.1111/add.16356

[CR44] Olderbak S, Rauschert C, Möckl J, Seitz N-N, Hoch E, Kraus L. Epidemiologischer Suchtsurvey 2021. Substanzkonsum und Hinweise auf substanzbezogene Störungen in Bayern, Nordrhein-Westfalen, Sachsen, Sachsen-Anhalt und in den Stadtstaaten Berlin, Bremen und Hamburg. Hg. v. IFT Institut für Therapieforschung. München; 2023. Available online https://www.esa-survey.de/fileadmin/user_upload/esa_laenderberichte/ESA-2021-Bundeslaenderbericht_2023-02-16_fin.pdf.

[CR45] Page RL, Allen LA, Kloner RA, Carriker CR, Martel C, Morris AA, et al. Medical Marijuana, recreational Cannabis, and cardiovascular health: A scientific statement from the American heart association. Circulation. 2020;142(10):e131–52. 10.1161/CIR.0000000000000883. 32752884 10.1161/CIR.0000000000000883

[CR46] Petzke F, Enax-Krumova EK, Häuser W. Wirksamkeit, Verträglichkeit und sicherheit von cannabinoiden Bei neuropathischen schmerzsyndromen: eine systematische Übersichtsarbeit von randomisierten, kontrollierten studien. Der Schmerz. 2016;30(1):62–88. 10.1007/s00482-015-0089-y. 26830780 10.1007/s00482-015-0089-y

[CR47] Radić M, Donner I, Waack M, Brinkmann C, Stein L, Radić D. Digitale gesundheitsanwendungen: die Akzeptanz Steigern. Deutsches Ärzteblatt. 2021;118(6):286.

[CR48] Rauschert C, Möckl J, Seitz N-N, Wilms N, Olderbak S, Kraus L. The use of psychoactive substances in Germany. Dtsch Arztebl Int. 2022;119:31–2. 10.3238/arztebl.m2022.0244. 35791270 10.3238/arztebl.m2022.0244 PMC9677535

[CR49] Rønne Sabrina Trappaud, Rosenbæk Frederik, Pedersen Line Bjørnskov, Waldorff Frans Boch, Nielsen Jesper Bo, Riisgaard Helle, et al. Physicians’ experiences, attitudes, and beliefs towards medical cannabis: a systematic literature review. BMC Fam Pract. 2021;22(1):212. 10.1186/s12875-021-01559-w. 34674661 10.1186/s12875-021-01559-w PMC8532330

[CR50] Rubin-Kahana DS, Crépault J-F, Matheson J, Le Foll B. The impact of cannabis legalization for recreational purposes on youth: a narrative review of the Canadian experience. Front Psychiatry. 2022;13:984485. 10.3389/fpsyt.2022.984485. 36213917 10.3389/fpsyt.2022.984485 PMC9539831

[CR51] Russo E, Agredano PM, Flachenecker P, Lawthom C, Munro D, Hindocha C, et al. The attitudes, knowledge and confidence of healthcare professionals about cannabis-based products. J Cannabis Res. 2024;6(1):32. 10.1186/s42238-024-00242-y. 10.1186/s42238-024-00242-y PMC11267914 39049083

[CR52] Sazegar P. Cannabis essentials: tools for clinical practice. Am Fam Physician. 2021;104(6):598–608. 34913644

[CR53] Schaefer Jonathan D., Hamdi Nayla R., Malone Stephen M., Vrieze Scott, Wilson Sylia, McGue Matt, et al. Associations between adolescent cannabis use and young-adult functioning in three longitudinal twin studies. Proc Natl Acad Sci U S A. 2021. 10.1073/pnas.2013180118. 33782115 10.1073/pnas.2013180118 PMC8040790

[CR54] Schmidt-Wolf Gabriele, Cremer-Schaeffer Peter. 3 Jahre Cannabis als Medizin – Zwischenergebnisse der Cannabisbegleiterhebung. Bundesgesundheitsblatt - Gesundheitsforschung - Gesundheitsschutz. 2021;64(3):368–77. 10.1007/s00103-021-03285-1. 33564897 10.1007/s00103-021-03285-1 PMC7932947

[CR55] Sharma Akash, Minh Duc Nguyen Tran, Luu Lam Thang Tai, Nam Nguyen Hai, Ng Sze Jia, Abbas Kirellos Said, et al. A consensus-based checklist for reporting of survey studies (CROSS). J Gen Intern Med. 2021;36(10):3179–87. 10.1007/s11606-021-06737-1. 33886027 10.1007/s11606-021-06737-1 PMC8481359

[CR56] Soos B, Garies S, Cornect-Benoit A, Montgomery L, Sharpe H, Rittenbach K, et al. Documenting cannabis use in primary care: a descriptive cross-sectional study using electronic medical record data in Alberta, Canada. BMC Res Notes. 2023;16(1):9. 10.1186/s13104-023-06274-6. 36726135 10.1186/s13104-023-06274-6 PMC9890680

[CR57] Sozialdemokratische Partei Deutschlands (SPD); Bündnis 90/Die Grünen; Freie Demokratische Partei (FDP), Herausgeber. Mehr Fortschritt wagen. Bündnis für Freiheit, Gerechtigkeit und Nachhaltigkeit. Koalitionsvertrag zwischen der Sozialdemokratischen Partei Deutschlands (SPD), Bündnis 90/Die Grünen und den Freien Demokraten (FDP). Bundesregierung. Berlin. Online verfügbar unter. 2021. https://www.spd.de/fileadmin/Dokumente/Koalitionsvertrag/Koalitionsvertrag_2021-2025.pdf, zuletzt geprüft am 09.10.24.

[CR58] Syed SA, Singh J, Elkholy H, Rojnić Palavra I, Tomicevic M, Eric A, Petek, et al. International perspectives on physician knowledge, attitudes, and practices related to medical cannabis. Front Public Health. 2025;13:1463871. 10.3389/fpubh.2025.1463871. 40051509 10.3389/fpubh.2025.1463871 PMC11882600

[CR59] Tait Margaret-Ann, Costa Daniel S. J., Campbell Rachel, Norman Richard, Warne Leon N., Schug Stephan, et al. Health-related quality of life in patients accessing medicinal cannabis in Australia: the QUEST initiative results of a 3-month follow-up observational study. PLoS One. 2023;18(9):e0290549. 10.1371/journal.pone.0290549. 37672515 10.1371/journal.pone.0290549 PMC10482296

[CR60] Wang L, Hong PJ, May C, Rehman Y, Oparin Y, Hong CJ et al. Medical cannabis or cannabinoids for chronic non-cancer and cancer related pain: a systematic review and meta-analysis of randomised clinical trials. In: BMJ (Clinical research ed.) 374, n1034. 2021. 10.1136/bmj.n1034. 10.1136/bmj.n1034 34497047

[CR61] Zammit Dimech David, Grech Louise, Serracino Inglott Anthony. Doctors’ and pharmacists’ perspectives on the clinical use of medicinal cannabis: a cross-sectional study. Harm Reduct J. 2025;22(1):167. 10.1186/s12954-025-01317-6. 41084005 10.1186/s12954-025-01317-6 PMC12519856

[CR62] Zentralinstitut für die Kassenärztliche Versorgung in Deutschland (Zi). Sondererhebung „Situation auf dem Arbeitsmarkt für MFA aus Sicht der niedergelassenen Ärzte und Psychotherapeuten für die Kassenärztliche Vereinigung Hansestadt Bremen (KVHB). Berlin; 2022. Available online, last accessed:16.05.2025. https://www.kvhb.de/fileadmin/kvhb/pdf/Umfragen/MFA-Umfrage-Ergebnis-2022.pdf.

[CR63] Zöllner C, Böhmer A, Geldner G, Karst J, Obertacke U, Pauschinger M et al. Präoperative Evaluation erwachsener Patientinnen und Patienten vor elektiven, nicht herz-thorax-chirurgischen Eingriffen. Eine gemeinsame Empfehlung der Deutschen Gesellschaft für Anästhesiologie und Intensivmedizin, der Deutschen Gesellschaft für Chirurgie und der Deutschen Gesellschaft für Innere Medizin. In: Anästhesiologie & Intensivmedizin (65), S. 2024:240–270. 10.19224/ai2024.240. 10.1007/s00101-024-01408-2 PMC11076399 38700730

[CR64] Zolotov Y, Mendoza Temple L, Isralowitz R, Gorelick DA, Abraham R, Abrams DI, et al. Developing medical cannabis competencies: a consensus statement. JAMA Netw Open. 2025;8(10):e2535049. 10.1001/jamanetworkopen.2025.35049. 41055914 10.1001/jamanetworkopen.2025.35049

[CR65] Zolotov Y, Metri S, Calabria E, Kogan M. Medical cannabis education among healthcare trainees: a scoping review. Complement Ther Med. 2021;58:102675. 10.1016/j.ctim.2021.102675. 10.1016/j.ctim.2021.102675 33539943

[CR66] Zolotov Y, Vulfsons S, Zarhin D, Sznitman S. Medical cannabis: an oxymoron? Physicians’ perceptions of medical cannabis. Int J Drug Policy. 2018;57:4–10. 10.1016/j.drugpo.2018.03.025. 29653439 10.1016/j.drugpo.2018.03.025

