# Supplementary material for: Attitudes and expectations of primary care physicians regarding recreational cannabis legalization in Germany: a pre-implementation survey
Source: J Cannabis Res. 2025 Dec 2;7:101. doi: 10.1186/s42238-025-00367-8 (PMC12690894; doi:10.1186/s42238-025-00367-8)
Supplement: Supplementary file 1 — Supplementary Material 1. [file 42238_2025_367_MOESM1_ESM.pdf]

5. Soziodemografische Daten

5.1 Welchem Geschlecht fühlen Sie sich zugehörig?

☐ männlich ☐ weiblich ☐ divers

5.2 Welcher Facharztrichtung gehören Sie an? *(Mehrfachnennung möglich)*

☐ Allgemeinmedizin

☐ Anästhesie

_______________ *Freitext*

5.2.1 Welche Zusatzweiterbildung haben Sie?

☐ Palliativmedizin ☐ keine

☐ Schmerzmedizin _______________ *Freitext*

5.3 Wie alt sind Sie?

_____ Zahlenwert

5.4 Seit wie vielen Jahren sind Sie im ambulanten Bereich tätig (inkl. Weiterbildungszeit)?

_____ Zahlenwert

5.5 Wie viele Stunden pro Woche arbeiten Sie im ambulanten Bereich?

☐ Vollzeit ☐ > 20 h ☐ ≤ 20 h

5.6 Wie viele Scheine generieren Sie pro Quartal durchschnittlich in der Praxis persönlich?

☐ < 100 ☐ 100 - 500 ☐ 501 - 1000 ☐ > 1000

5.7 In welcher Praxisform arbeiten Sie?

☐ Einzelpraxis ☐ Gemeinschaftspraxis ☐ Praxisgemeinschaft ☐ MVZ ☐ andere

Vielen Dank für Ihre Unterstützung und Mitarbeit!

Erfahrungen - Erwartungen der Cannabis Legalisierung

QR Code

Die Bundesregierung plant die Legalisierung von Cannabis.
Wenig bekannt sind Erfahrungen niedergelassener Hausärzte und Anästhesisten und deren Meinungen zu erwartbaren Folgen der Cannabislegalisierung. Dieser Fragebogen soll dazu beitragen, diese Wissenslücken zu schließen.

Haben Sie Patienten, die mit Ihnen über Cannabis sprechen? ☐ Ja ☐ Nein

1. Erfahrungen mit Patienten, die Cannabis konsumieren

(medizinischer Cannabis ausgenommen)

1.1 Wie häufig fragen Sie Ihre Patienten nach dem Konsum von Drogen *(außer Alkohol und Tabak)?*

Gar nicht ☐ ☐ ☐ ☐ ☐ ☐ Sehr häufig

1.1.1 Was ist der <u>häufigste</u> Anlass, an dem Sie Ihre Patienten nach Drogen fragen *(außer Alkohol und Tabak)?*

☐ in der Erstanamnese ☐ in jedem Quartal ☐ Ich frage nie. *(weiter bei 1.2)*

☐ beim Check up ☐ nach Bauchgefühl

☐ bei Verdacht/ Anhaltspunkten _______________ *Freitext*

1.1.2 Fragen Sie Ihre Patienten häufig explizit nach illegalen Cannabiskonsum?

Nein, gar nicht ☐ ☐ ☐ ☐ ☐ ☐ Ja, sehr häufig

1.2 Wie viele Patienten betreuen Sie schätzungsweise pro Jahr, die illegal Cannabis konsumieren?

☐ Weiß ich nicht ☐ Keinen *(weiter bei 2.1)* _____ Zahlenwert

1.2.1 Sprechen Sie mit Ihren Patienten, die Cannabis anwenden, über deren Cannabiskonsum?
(F1x.2= Substanzabhängigkeit, F1x.1= schädlicher Gebrauch)

☐ Ja ☐ Nein

2. Erfahrungen mit der Verschreibung von medizinischem Cannabis

2.1 Verordnen Sie in Ihrer Praxis medizinisches Cannabis?

☐ Ja

☐ Nein *(weiter bei 2.1.5)*

2.1.1 Welche Symptomatik wird am <u>häufigsten</u> in Ihrer Praxis mit medizinischem Cannabis behandelt? *(Einfachauswahl)*

Bitte nur eine Antwort!

☐ Schmerz
☐ Spastik
☐ Anorexie

.... *Freitext*

2.1.2 Wie hat sich die zuvor angegebene Symptomatik im Durchschnitt unter Therapie mit medizinischem Cannabis verändert?

☐ Deutlich verschlechtert
☐
☐ Keine Veränderung
☐
☐ Deutlich verbessert

2.1.3 Wie viele Ihrer Patienten erhalten schätzungsweise medizinisches Cannabis pro Quartal?

.... Zahlenwert

2.1.4 Wie viele Ihrer Patienten, die medizinisches Cannabis erhalten, konsumieren zusätzlich illegal Cannabis?

☐ Keine
☐
☐
☐
☐
☐ Sehr viele

☐ Weiß ich nicht.

2.1.5 Warum verordnen Sie kein medizinisches Cannabis? *(Mehrfachauswahl möglich)*

☐ Ich kenne mich nicht damit aus.
☐ Ich bin noch nie darum gebeten worden.
☐ Ich behandle keine Patienten mit entsprechenden Indikationen.
☐ Ich halte nichts von Cannabistherapie.
☐ Die Verordnung ist mir bürokratisch zu aufwändig.
☐ Es ist zu zeitaufwändig, mich umfassend mit der Therapie auseinander zu setzen.
☐ Ich habe Sorge, Suchtkrankheiten zu verstärken.
☐ ________________________________

____
____
____
____
____
____
____
____

3. Eigene Erfahrungen mit Cannabiskonsum

3.1 Haben Sie jemals wissentlich Cannabis konsumiert?

☐ Ja

☐ Nein *(weiter bei 4.1)*

3.1.1 Wie alt waren Sie, als Sie das letzte mal Cannabis konsumiert haben?

☐ 0 - 10 Jahre	☐ 21 - 30 Jahre	☐ 41 - 50 Jahre	☐ 61 - 70 Jahre
☐ 11 - 20 Jahre	☐ 31 - 40 Jahre	☐ 51 - 60 Jahre	☐ 71 - 80 Jahre

4. Erwartungen - Erwartete Folgen der Cannabislegalisierung

4.1 Wie schätzen Sie folgende Aussagen ein? Durch die Legalisierung von Cannabis

	Stark abnehmen		Keine Veränderung		Stark zunehmen	
... wird der Cannabiskonsum unter meinen Patienten ...	☐	☐	☐	☐	☐	
... wird die Zahl meiner Patienten mit behandlungs-bedürftigen Cannabiskonsumstörungen ...	☐	☐	☐	☐	☐	
... wird der Konsum meiner Patienten von anderen illegalen Drogen ...	☐	☐	☐	☐	☐	
... werden meine Verordnungen für medizinisches Cannabis ...	☐	☐	☐	☐	☐	○ Nicht zutreffend
... wird die Zahl der Patienten, die mir Fragen über Cannabis stellt ...	☐	☐	☐	☐	☐	

4.2 Wie schätzen Sie die Umsetzbarkeit der unten aufgeführten vier Ziele der deutschen Bundesregierung bezüglich der Cannabislegalisierung ein?

	Überhaupt nicht umsetzbar					Sehr gut umsetzbar
1) Die Qualität von Cannabis wird kontrolliert.	☐	☐	☐	☐	☐	☐
2) Die Weitergabe verunreinigter Substanzen wird verhindert.	☐	☐	☐	☐	☐	☐
3) Jugend- und Gesundheitsschutz für Konsumenten wird bestmöglich gewährleistet.	☐	☐	☐	☐	☐	☐
4) Der Schwarzmarkt wird eingedämmt.	☐	☐	☐	☐	☐	☐

4.3 Durch die Legalisierung von Cannabis

	Stimme gar nicht zu					Stimme voll zu
... werde ich mich zur kompetenten Cannabisberatung fortbilden.	☐	☐	☐	☐	☐	☐
