# Supplementary material for: Attitudes and expectations of primary care physicians regarding recreational cannabis legalization in Germany: a pre-implementation survey
Source: J Cannabis Res. 2025 Dec 2;7:101. doi: 10.1186/s42238-025-00367-8 (PMC12690894; doi:10.1186/s42238-025-00367-8)
Supplement: Supplementary file 2 — Supplementary Material 2. [file 42238_2025_367_MOESM2_ESM.pdf]

Experiences - Expectations of Cannabis Legalization

The federal government plans to legalize cannabis.

Little is known about the experiences of general practitioners and office-based anesthesiologists and their opinions on the expected consequences of cannabis legalization. This questionnaire aims to help close these knowledge gaps.

Do you have patients who discuss cannabis with you?

☐ Yes ☐ No

1. Experience with patients who consume cannabis (excluding medical cannabis)

1.1 How often do you ask your patients about drug use (excluding alcohol and tobacco)?

Not at all ☐ ☐ ☐ ☐ ☐ ☐ Very frequently

1.1.1 What is the most common occasion when you ask your patients about drugs (excluding alcohol and tobacco)?

☐ during initial medical history

☐ during check-ups

☐ in case of suspicion/indications

☐ in each quarter

☐ based on gut feeling

________________ (free text)

☐ I never ask. (continue to 1.2)

1.1.2 Do you frequently ask your patients explicitly about illegal cannabis use?

No, not at all ☐ ☐ ☐ ☐ ☐ ☐ Yes, very frequently

1.2 How many patients do you estimate you care for per year who illegally consume cannabis?

____________(numerical value) ☐ I don't know

☐ none (continue to 2.1)

1.2.1 Do you discuss cannabis use with your patients who use cannabis? (F1x.2= substance dependence, F1x.1= harmful use)

☐ Yes ☐ No

2. Experience with prescribing medical cannabis

2.1 Do you prescribe medical cannabis in your practice?

☐ Yes

☐ No (continue to 2.1.5)

2.1.1 Which symptoms are most commonly treated with medical cannabis in your practice?
(single choice)

Only one answer please

☐ Pain

☐ Spasticity

☐ Anorexia

☐ ________________ (free text)

2.1.2 How has the previously indicated symptom changed on average under treatment with medical cannabis?

☐ ☐ ☐ ☐ ☐

Significantly worsened	no change	Significantly improved

2.1.3 How many of your patients receive medical cannabis per quarter approximately?

______________ (numerical value)

2.1.4 How many of your patients who receive medical cannabis also consume illegal cannabis?

☐ ☐ ☐ ☐ ☐ ☐

None	Very many

☐ I don't know

2.1.5 Why don't you prescribe medical cannabis??
(Mehrfachauswahl möglich)

☐ I'm not familiar with it.

☐ I've never been asked for it.

☐ I don't treat patients with relevant indications.

☐ I don't believe in cannabis therapy.

☐ The prescription process is too bureaucratic for me.

☐ It's too time-consuming to thoroughly understand the therapy.

☐ I'm concerned about reinforcing addiction disorders.

☐ ___________________________(free text)

3. Personal experience with cannabis consumption

3.1 Have you ever knowingly consumed cannabis?

☐ Yes

☐ No

(continue to 4.1)

3.1.1 How old were you when you last consumed cannabis?

☐ 0-10 years	☐ 41- 50 years
☐ 11-20 years	☐ 51- 60 years
☐ 21- 30 years	☐ 61- 70 years
☐ 31- 40 years	☐ 71- 80 years

---

4. Expectations - Expected consequences of cannabis legalization

4.1 How do you assess the following statements. Through the legalization of cannabis...

	Strongly decrease		No change		Strongly increase	
...cannabis consumption among my patients will...	☐	☐	☐	☐	☐	
...the number of my patients with cannabis use disorders requiring treatment will...	☐	☐	☐	☐	☐	
...my patients' consumption of other illegal drugs will...	☐	☐	☐	☐	☐	
...my prescriptions for medical cannabis will...	☐	☐	☐	☐	☐	☐ not applicable
...the number of patients who ask me questions about cannabis will...	☐	☐	☐	☐	☐	

4.2 How do you assess the feasibility of the four goals of the German federal government regarding cannabis legalization listed below?

	Not feasible at all					Very feasible
1) The quality of cannabis will be controlled.	☐	☐	☐	☐	☐	☐
2) The distribution of contaminated substances will be prevented.	☐	☐	☐	☐	☐	☐
3) Youth and health protection for consumers will be ensured as best as possible.	☐	☐	☐	☐	☐	☐
4) The black market will be curtailed.	☐	☐	☐	☐	☐	☐

4.3. Through the legalization of cannabis

	Strongly disagree				Strongly agree
...I will seek further training for competent cannabis counseling.	☐	☐	☐	☐	☐

5. Sociodemographic data

5.1 Which gender do you identify with?

☐ male
☐ female
☐ diverse

5.2 Which medical specialty do you belong to? (Multiple selections possible)
☐ General practice
☐ Anesthesiology
____________________________________ (free text)

5.2.1 What additional qualifications do you have?
☐ Palliative Medicine
☐ Pain Medicine
____________________________________ (free text)
☐ none

5.3 How old are you?
____________________________________ (numerical value)

5.4 For how many years have you been working in the outpatient sector (including training period)?
____________________________________ (numerical value)

5.5 How many hours per week do you work in the outpatient sector?
☐ Full-time
☐ > 20h
☐ ≤ 20h

5.6 How many patient insurance cards do you personally process on average per quarter in your practice?

☐ <100
☐ 100- 500
☐ 501- 1000
☐ > 1000

5.7 In welcher Praxisform arbeiten Sie? In what type of practice do you work?
☐ Solo practice
☐ Group practice*
☐ Practice association**
☐ Medical care center
☐ other

Thank you very much for your support and cooperation!

* "Gemeinschaftspraxis" in German, i.e. doctors share patients, records, staff, equipment, and finances; they operate as a single legal and economic unit with shared liability.
** "Praxisgemeinschaft" in German, i.e. doctors share physical space and possibly some resources (waiting room, equipment, administrative staff) for cost efficiency, but maintain separate practices with their own patients, medical records, billing, and legal identity.
